# The use of Kumpfer’s resilience framework in understanding the breastfeeding experience of employed mothers after returning to work: a qualitative study in China

**DOI:** 10.1186/s13006-022-00459-8

**Published:** 2022-02-22

**Authors:** Honghua Guo, Rong Zhou, Minxiang Li, Siqi Zhang, Huanying Yi, Linjie Wang, Tong Li, Caihong Zhang, Hong Lu

**Affiliations:** 1 International Nursing School, Hainan Medical University, Haikou, China; 2 Department of Nursing, Second Affiliated Hospital of Hainan Medical University, Haikou, China; 3 School of Nursing, Peking University, Beijing, China

**Keywords:** Continuing to breastfeed, Employed mothers, Returning to work, Kumpfer’s Resilience Framework, China, Qualitative study

## Abstract

**Background:**

The increasing numbers of women in the workforce is an inevitable trend in China. More and more employed women stop breastfeeding because of working stressors. Many mothers, however, overcome the challenges and insist on breastfeeding after returning to work. Their individual experience of breastfeeding may provide a new insight to promote and support breastfeeding on employed mothers. This study sought to understand mothers’ experience with insisting on breastfeeding after returning to work based on Kumpfer’s Resilience Framework in Chinese context.

**Methods:**

This qualitative study was designed with semi-structured interviews. Purposive sampling and snowball sampling were employed to recruit 13 full-time working mothers with a stable job in the public sector who continued to breastfeed for 1 month or longer after returning to work in Haikou, Hainan Province, China. Interviews were conducted from January to March 2020 to capture participants’ experiences of breastfeed after returning to work. Grounded theory and Kumpfer’s Resilience Framework were used to analyze data via a systematic and iterative process.

**Results:**

Employed mothers built resilience while continuing to breastfeed after returning to work. The core concept was "dynamic interaction". Other categories were the background and explanation of this phenomenon. For working mothers who continued to breastfeed, resilience involved "dynamic interaction", which started from "experiencing stressors" and "obtaining support", two environmental factors interacted with the individual to "build resilience qualities", which interact with environment led to “behavioral resilience”. And then the ongoing dynamic interaction between behavioral resilience and environment ultimately led to three different "weaning processes", including natural weaning, active weaning, and forced weaning.

**Conclusions:**

This study identified the framework of resilience in mothers who were in the adversity of breastfeeding after returning to work based on Kumpfer’s Resilience Framework. It provided a new insight into the resilience of employed mothers around the world to continue breastfeeding and showed the different culture of breastfeeding on employed mothers.

## Background

Promoting breastfeeding is currently a major effort worldwide. The World Health Organization(WHO) recommends that exclusive breastfeeding is the best feeding method for infants up to 6 months after birth and suggests that mothers continue to breastfeed until their children are 2 years old or more [1]. The current situation of breastfeeding in China is less than satisfactory. The 2019 Report on Relevant Factors of Breastfeeding from the China Development Research Foundation(CDRF) shows that the rates are 29.2% for exclusive breastfeeding, 31.0% for breastfeeding with the addition of water or juice only, and 33.5% for mixed feeding during the first 6 months after birth in China. The rate of any breastfeeding decreases to 66.5% when the infant reaches 11 months old. Among employed women, returning to work is one of the main reasons for the decline in breastfeeding after 6 months. This is a common challenge for working mothers around the world [2, 3].

In 2019 in China, a developing country, 60.5% of women participated in the labor force, the highest percentage in the Asia–Pacific region [4]. According to the newest Chinese maternity leave policy in 2012, employed women have maternity leave of 98 days after childbirth [5]. When they return to work, employers should arrange one hour for breastfeeding during daily work and set up a breastfeeding room as needed [5]. However, according to CDRF (2019) survey results, only 67.2% employed mothers in China could have one hour of breastfeeding time a day and 19.1% had breastfeeding room in their workplaces, and 49.0% of them had public refrigerators to store their breast milk [2]. These indicated that working conditions for most mothers returning to work in China cannot meet employed women’s breastfeeding needs. Therefore, these mothers have to handle both work and breastfeeding and face personal, institutional, and social pressure and challenges. For example, they face challenges such as an inflexible work schedule, a restrictive work environment, short maternity leave, and a lack of special maternity rooms, private milk-expressing space, and milk-expressing time in the workplace [6, 7]. Some women make a personal decision to end breastfeeding after childbirth due to a lack of knowledge on the topic. Working stressors, peer pressure and lack of family support are other reasons for working women to stop breastfeeding [8, 9]. A study in Shanghai, China, shows that the rate of breastfeeding is as low as 17.5% in employed mothers after 12 months [10]. Therefore, attention may be paid to breastfeeding among employed mothers in economically developed areas of China.

Although working stressors and challenges cause more and more employed mothers to stop breastfeeding in China, the large national survey conducted by CDRF shows that there are still 81.3% of employed women continue to breastfeed by going home to breastfeed at work, taking pumped breast milk home, breastfeeding at the workplace, and breastfeeding at home after work [2], suggesting that they have overcome many difficulties and are resilient despite adversity [11]. Sulaiman et al. have showed that employed mothers in Malaysia experienced a similar working environment may have the different outcomes of breastfeeding because their characteristics were different, ‘passionate’ mothers with a strong determination can insist on exclusive breastfeeding for 6 months more easily [12]. The researchers also found women’s belief and perception of breastfeeding was more important than the timing of returning to work in maintaining breastfeeding [13]. Johnsen (2002) mentioned the concept of resilience in breastfeeding for the first time. She used her own experience to discover that women can resist stress and insist on breastfeeding. So she conducted interviews with some breastfeeding women of low incomes and found that these employed mothers possess the qualities of resilience [14]. What are the characteristics and perception of Chinese employed mothers with insisting on breastfeeding after returning to work? How do they overcome the challenges and stressors? We used Kumpfer’s Resilience Framework to understand the experience of mothers who insisting on breastfeeding after returning to work in order to answer these questions. The framework believes that environmental stimuli break the previous balance, lead to the interaction between the environment and the individual, enhance the characteristics of resilience, and then make the characteristics of resilience interact with environmental dynamics[15].This study aims to learn about mothers’ experience with insisting on breastfeeding after returning to work from the perspective of resilience and to understand how they build and promote resilience in this process, in order to provide new insights and ideas for related studies to promote breastfeeding among employed women in China.

## Theoretical framework

Kumpfer’s Resilience Framework was employed as the theoretical framework in this study because it believes that stressors and challenges are the stimulus for resilience and internal resiliency characteristics are to grow under pressure [15]. It focuses on the dynamic interaction between environmental factors and individuals. According to this theoretical framework, the environmental stimulus breaks the previous balance and leads to interactions between the environment and individual, which strengthens resilience characteristics, and then resilience characteristics interact with environment dynamically. Finally, three outcomes appear such as resilience reconstruction, adaptation and maladaptive reconstruction [15, 16]. This framework was first used to describe resilience process in adversity in children and adolescents [17] and was later used in experiential research in caregivers of disabled elderly people [18] and resilience experiential research in patients with special conditions such as cancer and their families [19, 20]. This study used Kumpfer’s Resilience Framework to learn about resilience in employed mothers who continue to breastfeed after returning to work despite challenges.

## Methods

### Study design

This was a qualitative study. Employed mothers who continued to breastfeed for 1 month or more after returning to work participated in a semi-structured interview to talk about their experience, challenges and stressors, and efforts to overcome the challenges. We used Kumpfer’s Resilience Framework as the theoretical framework for the study design and data analysis to help us understand how employed women continued to breastfeed despite challenges.

## Study setting and participants

This study was conducted in Haikou, the capital city of Hainan Province, China. Haikou, a developing island city, is located at the southernmost tip of China. As in other cities in China, as the female labor force participation rate has risen, more and more local women of childbearing potential are working, especially in the public sector with job security [21, 22]. In this study, we used purposive sampling and snowball sampling to recruit full-time working mothers with a stable job in the public sector who continued to breastfeed for 1 month or more after returning to work and agreed to participate in the interview. The study identifies working mothers based on breastfeeding time and occupational nature. The investigators considered the duration of breastfeeding after returning to work and the occupation of each working mother to ensure the adequacy and diversity of the information the participants would give. The sample size was set based on information saturation [23].

## Data collection

The semistructured interview guide was developed based on Kumpfer’s Resilience Framework and on the study purpose. Initially, a pilot study was conducted with two employed mothers to evaluate the guiding questions. The topics included basic information; the mothers’ experience of breastfeeding after returning to work; individual difficulties and stressors in balancing personal life, breastfeeding, and work; stress responses and management strategies; and efforts to overcome challenges. Most interview questions were open-ended. Two experienced qualitative researchers of the study team conducted semi-structured interviews from January to March 2020 to collect data. The appointment was set up via telephone, and the location was selected by the participant. Thirteen participants were interviewed in their own familiar settings (bedroom, office, conference room at work, etc.). During the interview, the researcher observed nonverbal behaviors of interviewees to deepen understanding on the interviewees. Each interview lasted 40 to 60 min. Interview recordings were compiled and transcribed and then imported into Excel by the interviewer soon after each interview. A copy of the text was provided to the interviewee to ensure accuracy.

## Data analysis

In this study, we combined grounded theory coding and the theoretical framework and collected and analyzed data via a systematic and iterative process. According Strauss and Corbin (1998), the procedure of ground theory coding includes three levels of coding: open coding, axial coding and selective coding [23]. To ensure the reliability of the analysis, the data analysis started from the time of data collection, and each transcribed original text was manually analyzed in Excel. Two members of the study team independently reviewed the data to identify any open code. During axial and selective coding, the study team met regularly to discuss and develop axial codes through continuous comparison to identify the core categories, which, along with Kumpfer’s Resilience Framework, were used to finalize the new concept and theme framework for characterizing resilience among employed mothers who continued to breastfeed [23, 24]. Throughout the process, the study team supported the work by ongoing memos and tables [25]. The study team also revised interview questions, developed topics, and monitored information saturation as needed until the end of data collection.

## Trustworthiness of data

To ensure the trustworthiness of our data, we continuously reflected how to reduce the impact of subjectivity on the results [26]. we did a great deal of literature reviews and kept abreast of literature updates during the data collection and data analysis. The two researchers interviewed in this study have 13 and 5 years of qualitative research experience, and they have no personal experience of breastfeeding, which can avoid the subjective influence of personal experience to a certain extent. However, it may lead to a loss of empathy during the interview, more or less.

During the interview, we tried to keep neutral attitude to communicate with the interviewee [27]. We paid attention to avoid using our own opinions to influence the interviewee, so as to obtain the real feelings and experience of working mothers as much as possible. Meanwhile, we observed the non-verbal behavior of the interviewee and made on-site notes to understand the surface and deep meaning of the interviewee's body language. Additionally, we provided the interview transcript to the participant for comments and confirmation. We also adopted the peer review technique among the researchers in reaching an agreement during the process of coding. All these methods were applied in this study to ensure the trustworthiness of data [28].

## Ethics

This study was approved by the Ethics Committee of the Second Affiliated Hospital of Hainan Medical University in December 2019. A signed informed consent form was obtained before interview. The investigators maintained interview audio recordings, which were destroyed at the completion of data analysis. The information stored in the computer was password-protected.

## Results

A total of 13 working mothers with different occupations, ages, educational levels, parities, times returning to work, and breastfeeding durations participated in this study. (Table 1) As for their occupations, there are four policewomen, three nurses, three teachers, a doctor, a designer and an accountant.

**Table 1:** Characteristics of mothers in the qualitative study

Classification		Number
Age range	20–30 years	3
	31–40 years	9
	> 40 years	1
educational level	Diploma level	1
	Bachelor's degree level	8
	Master degree level	4
Number of children in the time of interview	primiparous mother	5
	parous mother	8
Time of returning to work after birth	6 months	7
	> 6 months	6
Duration of breastfeeding	1 year	2
	> 1 year	11

The results show that mothers developed resilience while continuing to breastfeed after returning to work. The core concept was "dynamic interaction". Other categories were the background and explanation of this phenomenon. For employed mothers who continued to breastfeed, resilience involved "dynamic interaction", which started from "experiencing stressors" and "obtaining support", the two kinds of environmental factors that interacted with the individual to "build resilience qualities", which interact with environment to led to “behavioral resilience”, and then the ongoing dynamic interaction between behavioral resilience and environment ultimately led to three different "weaning processes".^1^ Table 2 summarizes the main categories identified in this study and the elements of each category.

**Table 2:** Summary of the categories of the study

**Dynamic interaction**													
**Experiencing stressors**			**Obtaining support**			**Resilience building**			**Behavioral resilience**		**Weaning processes**^**1**^		
**Personal pressure**	**External pressure**	**Self support**	**Family** **support**	**Workplace** **support**	**Rolemodels**	**Traits**	**Enhanced belief:**	**Deepened** **understanding** **of their role**	**Willingness** **to change**	**Selfadjustment**	**Natural** **weaning**	**Active** **weaning**	**Forced** **weaning**
dual role, relapse of old condition and dwindling of milk	family and social doubt,lack of spousal support, distance and economic pressure	began to learn and confidence in milk production	The importance of maternal grandmothers	Peer understanding, a reduced workload, and external support	Family members, colleagues, internet acquaintances	tenacity, perseverance, endurance	"give the child the best" and "breastfeeding to ensure a healthy child	a heightened sense of responsibility and natural instincts and a closer mother–child bond	“giving up oneself” and learning new information/skills	Feeding methods adjustment, milk production adjustment and schedule adjustment			

## Experiencing stressors

For employed mothers, their social roles have been changed. Being a working mother involved a myriad of new stressors and challenges, including personal and external stressors.

## Personal stressors

Mothers must manage a workload while taking the time to express and store milk. The dual role, relapse of illness and dwindling of milk may lead to physical and mental exhaustion. A mother who was a nurse intern said:

"Work is busy, with many exams, and it takes long time spent to express milk…. I use an electric breast pump to express milk at work, which is time-consuming…. At 1 a.m., in early morning…. Do you know how tiring it is to breastfeed? I am very tired. It takes so much energy and time, which makes it difficult to focus on work." (Mother1, breastfed for 10 months, Nurse intern).

Prolonged physical and mental exhaustion caused some old conditions to relapse in some mothers, which may affect milk production and thus the willingness to continue to breastfeed.

"He is restless when hungry. After spending 2 to 3 months taking care of him around the clock, including several times at night, my cervical condition started to bother me again…." (Mother2, breastfed for 10 months, Pediatric nurse).

"I have many stresses at work…. Milk production starts to dwindle over time, which affects my confidence and willingness to continue to breastfeed." (Mother3, breastfed for 18 months, Financial Accountant).

## External stressors

For employed mothers, family and social doubt was the first external stressor, which caused mental stress and reflected the current lack of social support for breastfeeding in China.

"My mother-in-law often tells me that I do not produce enough milk and that my milk is water-like with too little nutrition. This causes me to doubt my ability to feed my child." (Mother4, breastfed for 16 months, Policewoman).

"Sometimes when breastfeeding in public, I hear people talking about why such an ‘old’ child is still breastfeeding and why I am still not weaning my child from breastfeeding? I was very upset at these comments." (Mother5, breastfed for 17 months, University Teacher).

"My colleagues sometimes say, ‘why are you still breastfeeding and not weaning? It affects your work." (Mother3, breastfed for 20 months, Financial Accountant).

Lack of spousal support was another stressor. A mother said, "My husband often says that I know more about breastfeeding than he does, as if women should shoulder all the responsibilities in raising a child" (Mother6, breastfed for 14 months, Maternity nurse). This attitude made working mothers feel "upset and angry" (Mother6, breastfed for 14 months, Maternity nurse). It also reflected the traditional Chinese notion that "women should be responsible for raising children".

The distance between home and work and the economic status also posed challenges to working mothers:

"At the time, my home was far away from my workplace…. I personally breastfed my child. I was busy at work, while thinking about how to rush back home to breastfeed my child. The stress affected milk production, which was the biggest challenge."(Mother4, breastfed for 16 months, Policewoman).

"Given the economic problems at home, returning to work was my only option."(Mother2, breastfed for 10 months, Pediatric nurse).

## Obtaining support

Working mothers faced stressors and challenges when continuing to breastfeed, but they were also inspired by personal, family, and workplace support and role models. The level of support varied with the individual conditions.

## Self-support

Some mothers began to learn about breastfeeding through maternity check-up bulletin boards, brochures, and baby products starting when they were pregnant, which helped them to “understand more about breastfeeding”(Mother3, breastfed for 20 months, Financial Accountant), "strengthen my determination to continue to breastfeed (Mother2, breastfed for 10 months, Pediatric nurse)", and "take action to continue to breastfeed despite being tired and even though it was time-consuming because of my desire to keep the child safe and well nourished" (Mother1, breastfed for 10 months, Nurse intern).

Confidence in milk production was the source of confidence and motivation for continuing to breastfeed among working mothers. "The key is having enough milk.”(Mother1, breastfed for 10 months, Nurse intern), and "good milk production gives me the confidence to insist on breastfeeding" (Mother3, breastfed for 20 months, Financial Accountant).

## Family support

Based on Chinese tradition, most infants are taken care of by elderly family members who live with the parents after the mother returns to work. The active participation, encouragement, and affirmation of family members help working mothers to manage stress and be confident about breastfeeding. In particular, female elders with childbearing experience can pass their breastfeeding experience to working mothers, which strengthens their confidence in continuing to breastfeed. This also reflects the Chinese culture of family and breastfeeding will pass on by generations.

"My mother and mother-in-law put aside their own family and life to support me…. I did not need to do anything except go to work and breastfeed…. They often made soups to help me produce milk."(Mother1, breastfed for 10 months, Nurse intern).

"Whenever I talked to her [my mother] about weaning, she would tell me that it is not good for the development or health of my child....(Mother3, breastfed for 20 months, Financial Accountant)"

"I am confident. She [my mother] believes in me, giving me the confidence to insist on breastfeeding." (Mother7, breastfed for 15 months, Primary School Teacher).

## Workplace support

Workplace support came from peer understanding, a reduced workload, and other external support, which mitigated the negative effect of the conflict between working and breastfeeding.

An employed mother who was a teacher mentioned:

"Whenever I was absent when they did roll-call…they understood, and my boss was fine with it too. My colleagues understood that I was busy, and they were encouraging and appreciating my efforts." (Mother7, breastfed for 15 months, Primary School Teacher).

A working mother who was a mechanical designer recalled:

"Unlike some other jobs that involve urgencies or emergencies, my job environment is stable…we rarely work overtime…with no particular stress."(Mother8,breastfed for 10 months,Mechanical Designer).

Two workplaces had refrigerators to store breast milk (Mother2, breastfed for 10 months, Pediatric nurse; Mother1, breastfed for 10 months, Nurse intern), one woman got a one-hour breastfeeding break per day (Mother7, breastfed for 15 months, Primary School Teacher), and one woman’s workplace was close to home, all of which made it easier to go home to breastfeed (Mother9, breastfed for 18 months, Policewoman), made working mothers feel that their breastfeeding time was "sufficient"(Mother7, breastfed for 25 months, Primary School Teacher), and made breastfeeding "simple" (Mother2, breastfed for 10 months, Pediatric nurse).

## Role models

Some working mothers looked to role models who continued to breastfeed for how to manage stressors and challenges and to become more confident about breastfeeding. These role models were family members, colleagues, or internet acquaintances.

"My sister-in-law breastfed for two years. I do not want to wean too early. I believe that I can…."(Mother7, breastfed for 15 months, Primary School Teacher).

"A head nurse in our hospital breastfed for 18 months. And her children at the time were generally healthier… I admire her." (Mother6, breastfed for 14 months, Maternity nurse).

"I followed the public account of the La Leche League International. Those children were 5 or 6 years old and were still breastfeeding. I admire that…. Whenever I have challenges, I search for what other mothers are doing, which is very helpful." (Mother4, breastfed for 16 months, Policewoman).

## Resilience building

Adversity inspires excellent personal qualities [29]. Employed mothers who insisting on breastfeeding after returning to work by managing stressors and obtaining support demonstrated some special traits, strengthened belief in breastfeeding and deepened understanding of their roles.

## Traits

Working mothers who continued to breastfeed demonstrated tenacity, perseverance, and endurance, which were closely related to their personal experience within their family and the personalities of their parents. When facing challenges and hardship, working mothers wanted to "persevere in whatever [they were] doing” (Mother6, breastfed for 14 months, Maternity nurse).

"I had some challenges…during breastfeeding, but I was persistent…and resilient…. My mother taught me when I was little that no matter how difficult the situation is, I must persevere in whatever I am doing…."(Mother10, breastfed for 16 months, University Teacher).

"Negative comments from others only serve to strengthen my belief and actions…. I am as stubborn as my dad, and I will persevere in whatever I want to do, regardless of whether others agree." (Mother5, breastfed for 17 months, University Teacher).

"I continued to breastfeed for the sake of my child…. I was working and needed to take care of sick elderly family members, but I persisted". (Mother1, breastfed for 10 months, Nurse intern).

## Enhanced belief

Belief refers to the behavioral tendency of one’s own ideas, thoughts, and awareness, which is demonstrated as strong and unwavering conviction and trust [30]. The instinct to "give the child the best" and the pursuit of "breastfeeding to ensure a healthy child" were strengthened among the working mothers by their breastfeeding knowledge and their mother–child interactions, along with personality traits such as tenacity, perseverance, and endurance.

"I knew before returning to work that breast milk is life-giving and…precious, just like our child…." (Mother2, breastfed for 10 months, Pediatric nurse).

"Formula is a substitute, not a necessity. The nutritional value of formula is inferior to that of breast milk…. Breast milk is derived from *qi* and blood…. It is healthy…." (Mother11, breastfed for 16 months, Policewoman).

"When I reunite with my child after work, his dependence on me reinforces that I am very important in his life, which strengthens my belief that I must give him the best." (Mother7, breastfed for 15 months, Primary School Teacher).

"I tried to feed other food to my child, but she refused even when she was hungry… She wanted my milk… She looked so helpless, and I was determined…to keep going."(Mother4, breastfed for 16 months, Policewoman).

## Deepened understanding of their roles

The employed mothers faced the dual stresses of work and motherhood, but their children’s dependence on them deepened their understanding of motherhood, which promoted personal growth, leading to a heightened sense of responsibility and natural instincts and a closer mother–child bond.

"After returning to work, my child was still waiting for my milk. Whenever I was back home from work, I felt that I had the obligation to take care of him… Regardless of what happens, I must leave early…and go home to breastfeed…." (Mother7, breastfed for 15 months, Primary School Teacher).

"Once I was used to work-related stress, breastfeeding became a habit, just like having meals…. It's a natural female instinct." (Mother1, breastfed for 10 months, Nurse intern).

"During breastfeeding, we looked at each other and time just flew. I enjoyed the experience, although it was hard sometimes." (Mother10, breastfed for 16 months, University Teacher).

"I continued to breastfeed because I wanted to strengthen the mother–child bond…. I think it is difficult to guide and educate her when she gets older, if we do not develop basic intimacy between the two of us…." (Mother3, breastfed for 20 months, Financial Accountant).

## Behavioral resilience

For working mothers, resilience building was followed by behavior adjustment to manage stress in order to continue to breastfeed. This involved two phases, willingness to change and self-adjustment.

## Willingness to change

Some participants who continued to breastfeed were willing to "give up oneself" (Mother7, breastfed for 15 months, Primary School Teacher) to make time for breastfeeding. Mother3 recalled:

"… I gave up something, such as freedom…to have more time with my child…and for breastfeeding." (Mother3, breastfed for 20 months, Financial Accountant).

Some mothers began to learn new things from professionals, such as the causes of reduced milk production and how to improve milk production, so they could plan their breastfeeding times and take actions to improve milk production.

"…I attended an online training course…. It said that breast milk is rich in nutrition and immune factors…. It promotes mother–child bonding and improves the child’s self-confidence and behavior…. I initially planned to breastfeed for 10 to 12 months. After the course, I decided to breastfeed longer." (Mother3, breastfed for 20 months, Financial Accountant) It also means the timely and available professional help may promote the mothers to "change" and then prolong the time of breastfeeding.

## Self-adjustment

For some working mothers, self-adjustment is the manifestation of resilience in times of challenges. This includes the adjustment of feeding methods, milk production, and breastfeeding schedule. Some working mothers living far from their workplaces chose to express milk (Mother1, breastfed for 10 months, Nurse intern). Some had their family take their child to their workplace for breastfeeding (Mother2, breastfed for 10 months, Pediatric nurse). Some simply adjusted their feeding methods.

"After returning to work, I expressed milk during the day. If I had time, I went back home to breastfeed my child at lunch time…. Once in the evening, once in the morning, that was enough…." (Mother8, breastfed for 10 months, Mechanical Designer).

Some working mothers had problems with milk production, and they tried different methods to remedy the issue. Two mothers tried herbs, two mothers increased their soup intake to improve milk production and confidence, and one of them consulted breastfeeding professionals.

In the case of any conflicts between work and breastfeeding, some working mothers talked to their bosses about a flexible work schedule. "I talked to my manager…he agreed that I may come to work at 9:00 a.m. (no later than 9:30 a.m.) …for field work, I talked to my manager to see if someone else could stand in for me…." (Mother3, breastfed for 20 months, Financial Accountant).

## Weaning processes

As breastfeeding went on, working mothers continued to have new stresses as well as support. Their resilience continued to interact with environmental factors and led to new behavioral resilience to ensure continued breastfeeding. Finally, three weaning processes were identified such as natural weaning, active weaning, and forced weaning. The state and emotions of the mother and child varied between scenarios.

## Natural weaning

These working mothers enjoyed breastfeeding and were happy about motherhood. They also believed that they met the physical and mental needs of their child after a long time of breastfeeding.

"I enjoyed breastfeeding…. I did it for 16 months before weaning naturally…. At the time of weaning, I believed that my child no longer needed my milk." (Mother10, breastfed for 16 months, University Teacher).

"Milk preparation was not that hard or tiring. Weaning was natural, and I feel my child was satisfied and happy…." (Mother11, breastfed for 18 months, Policewoman).

## Active weaning

As breastfeeding went on, some working mothers faced changes in workload. They wanted to resume a normal workload before breastfeeding and adopted active weaning after a time of breastfeeding. The mental needs of their child may or may not have been met.

"Work was getting busier, my child stopped gaining weight…. I had breastfed for 10 months and believed that breast milk was no longer enough to meet his needs. It was time to stop breastfeeding." (Mother1, breastfed for 10 months, Nurse intern).

"I wanted to devote myself to my work as soon as possible."(Mother12, breastfed for 13 months, Policewoman).

"I planned to breastfeed for 12 months… Once the goal was achieved, it was time to stop breastfeeding, mission accomplished…." (Mother13, breastfed for 18 months, Deputy Chief Physician).

Some working mothers were "hesitant" about weaning (Mother6, breastfed for 14 months, Maternity nurse), but they felt the stressors outweighed support. "Milk volume started to decline, nighttime was difficult, both my husband and I were tired, I decided it was time to stop breastfeeding…. Then my child was a little sick and caught a cold. I wanted to resume breastfeeding…. But my family disagreed. They said that I had stopped breastfeeding, why restart it?… My child liked to touch my nipples while sleeping. He may have unmet mental needs." (Mother6, breastfed for 14 months, Maternity nurse).

## Forced weaning

Some employed mothers continued to breastfeed for a long time and faced more stressors than support as well as forced weaning. In this case, both mother and child had unmet mental needs. For Parous mother who breastfed for 10 months, her cervical condition worsened after returning to work, and her family opposed breastfeeding.

"You don’t have milk for the child, and he is restless… It may be better off to use formula feeding instead of breastfeeding…. I had no choice but to wean him from breastfeeding…. After weaning, whenever he saw me, he just kept crying…and wanted me to hug him."(Mother2, breastfed for 10 months, Pediatric nurse).

"Sometimes when we went out to play, we saw other children of a similar age…. I heard others comment about a child of 18 months old who was still breastfeeding…. I felt guilty…." (Mother2, breastfed for 10 months, Pediatric nurse).

## Dynamic interaction

**Fig. 1:**
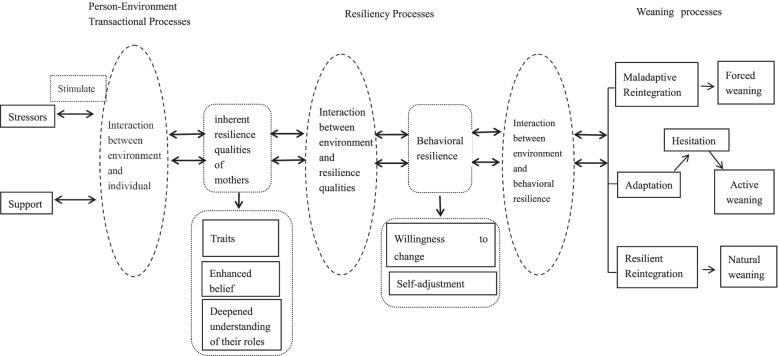
The framework of dynamic interactive resilience on employed mothers who continue to breastfeed after returning to work

In this study, the core category was "dynamic interaction". Employed mothers faced stressors and support, and their dynamic interaction with these environmental factors stimulated their inherent resilience qualities, which continued to dynamically interact with the environmental factors, leading to behavioral resilience. Behavioral resilience also interacted with environment dynamically and resulted in three different weaning processes. In brief, the identification of relationships of these themes led to the resilience model for working mothers who continued to breastfeed based on Kumpfer’s Resilience Framework (Fig. 1).

## Discussion

Women returning to work need to juggle stressors from work, baby care, family and individual to maintain breastfeeding. In this adversity of breastfeeding, our study found that they built the resilience and formed to three resilience outcomes by the dynamic interaction between environment and individuals. Figure 1 demonstrated the resilience model for working women who continued to breastfeed. This model included five predictors of resilience in breastfeeding women returning to work, namely stressors and support, person-environment transactional processes, resilience building, behavioral resilience and weaning processes.

## Stressors and support as environmental factors that working mothers face when they continue breastfeeding

According to the previous studies, shorter maternity leave, less supportive workplace environment and less social support are the barriers for employed mothers to maintain breastfeeding after returning to work, and they may lead to the end of breastfeeding for mothers [8, 9, 31]. In our study, the participants also experienced the similar barriers refer to personal, family, workplace, and social stressors, such as dual roles, social and family doubt, lack of support, relapsed medical conditions, childcare, inadequate milk volume, a long commute, and economic pressure. These stressors were identified the initiating environmental factors of resilience as suggested by Kumpfer’s Resilience Framework [10]. The results of this study showed that insufficient milk is one of the physiological stresses encountered by working women, which was also the main reason that Gunn believed that women wean (Gunn 1984) [32]. However, in our study, working women try to use various methods to increase lactation for the health of their babies, such as soup food therapy, while most women in a study conducted by Ellis in Canada would stop again because they felt insufficient breast milk (Ellis, 1984) [33]. This is mainly related to the difference in food culture and puerperal culture between China and Canada. Chinese women will confinement after childbirth, which means that pregnant women take one month (sequential delivery) or 42 days (cesarean section) to recuperate after delivery. The period of confinement is a critical period to assist the mothers to smoothly pass through the physiological and psychological transitions in their lives. It is a behavioral culture with a sense of Chinese ritual. During confinement, Chinese women will have a special diet therapy to increase lactation according to Chinese traditional culture. They may inspire the resilience quality rather than simply ending to breastfeed [15].

Our results show that working women also have protective factors of resilience include the support from themselves, their family, their workplace, and role models [15]. As found by Gilmour et al. (2013) flexibility, proximity and communication are important factors to balance the interactions between the workplace, family and baby care and promote to continue to breastfeed as long as possible [34]. Thus identifying the protective factors of resilience in employed mothers returning to work is essential. Specifically, most of the participants in this study highlighted the significant supportive role of maternal grandmothers on working women’s breastfeeding rather than husbands. This is different from other studies which show that fathers have more positive attitudes on mother’s breastfeeding than the maternal grandmother [35, 36]. It reflects various social, cultural factors on breastfeeding of working mother and suggests that fathers may need more professional support about breastfeeding. Besides, this study identified the protective factors of themself, family, workplace and peers, but the support from the policy, institutions, lactation consultants and health professionals were rarely mentioned by these employed mothers. This is in line with the views of Xiong et al. (2021) stating that there may be insufficient professional support for Chinese breastfeeding mothers [37]. At present, evidence of promoting breastfeeding in many countries has shown that support provided by medical institutions, health professionals or lactation consultants is the key to enhance the duration of breastfeeding [37, 38]. Rollins et al. (2016) believe that women's breastfeeding to a large extent depends on the support from their living community and environment [3]. Therefore, the government, institutions and health professionals need to take the responsibility to make regulations and plans to support employed mothers in breastfeeding. Their support may be the important protective factor of the resilience.

Some environmental factors are very individualized and complicated for employed mothers in our study. Different mothers had different stressors and supports from their families, workplaces, peers and themselves. Some of stressors and supports somehow may exchange each other dynamically. In our study, family members may be the support factors for some employed mothers during breastfeeding, but it may be stressors for other mothers. In the dynamic interaction between individual and her environmental factors, family member may be shifted from stressor to support in certain circumstances. Other environmental factors of workplaces, individuals, peers, the amount of breast milk, distance between home and workplace have the same feature in this study. Sulaiman et al. (2015)also revealed that workplace environment, human factors and individual characteristics could be barriers or enablers to mothers’ ability to sustain breastfeeding after returning to work [13]. Therefore, it requires midwives to assess employed mothers individually and continuously during their breastfeeding in order to identify which factors they need to moderate to promote mothers’ resilience to continue breastfeeding.

## Person-environment transactional processes stimulate the resilience building

According to Kumpfer’s resilience framework, person-environment transactional processes refer to internal interactions that individual would have when he or she consciously or unconsciously modifies environmental factors under pressure [15]. In this study, it showed in the process of obtaining support consciously. When facing stressors, some mothers talked to family members about their difficulties, while others might start to learn something new or seek to get support from their role models. During the processes, employed mothers tended to integrate their stress and support resources, and this integration also reenergized their internal self traits (such as tenacity, perseverance and endurance). Meanwhile, their internal self traits obtained further growth [16]. This growth found in our study promoted the enhanced belief of "giving the child the best" and "breastfeeding to ensure a healthy child", and then mothers acquired a heightened sense of responsibility, natural instincts and a closer mother–child bond. Night breastfeeding will increase the pressure of continuous breastfeeding of working women, which is mainly related to lack of energy during the day due to scattered sleep time. Dungy (1992) believed that women who received breast milk pumps would make it easier for them to breastfeed at night and prolong breastfeeding time [38]. In this study, employed mothers who were stressed by breastfeeding at night received support from their loved ones, and could develop tenacity and patience personal qualities and strengthen breastfeeding beliefs under pressure. The difference between the two studies may be related to their sociocultural background. The working women in this study were deeply influenced by traditional Chinese culture, and the family culture of "being strong for mothers" in women’s mothers will continue to influence them. Although Sulaiman et al. (2015)identified the interaction between working mothers’ personal attributes, the enablers and barriers based on Work-Family Conflict Framework and highlighted ‘passionate’ mothers with strong beliefs and intention could overrule other factors to maintain breastfeeding [13], our result suggested the growth of women’s internal self traits may be more important for the women to insist on breastfeeding after returning to work. This founding was consistent with Carey et al. (1998) mentioned that resilience can be improved over time [40].

Additionally, the resilience characteristics of internal self traits, enhanced belief and deepened understanding of their roles identified in this study are different from Kumpfer’s perspective seven resilience qualities including happiness, wisdom and insight, humor, empathy, intellectual competencies, purpose in life and perseverance [15]. Kumfer’s framework was used in children and adolescents originally [17] but this study targeted working mothers who continue to breastfeed after returning to work, which added the new perspective of resilience among different groups.

## Behavioral resilience is a process in which the resilience qualities and the environment continue to interact dynamically

After the processes of resilience building, the resilience qualities continued to interact with environment dynamically, which motivated employed mothers to take actions to manage stress in order to continue breastfeeding [16, 41]. Our results showed there were two phases of behavioral resilience, i.e. willingness to change and self-adjustment. In this process, working mothers tried to shift the high-risk environment into protective environment dynamically by changing their cognition, actively coping with difficulties, and making plans of breastfeeding time [15]. Through some problem-solving skills and actions taken, there would be different resilience outcomes.

## The process of behavioral resilience leads to three different weaning processes

Behavioral resilience interacted with environment ultimately led to three different outcomes (resilience reconstruction, adaptation, or maladaptive reconstruction) [15], which were consistent with the three weaning processes we mentioned in the results. Eventually every breastfeeding baby weaned. Relevant researches showed returning to work was the common cause of weaning (41%) but limited studies explained the weaning experience of employed mothers [42].

Natural weaning is a type of resilience reconstruction. Resilience reconstruction refer to a good interaction between individual resilience and the environment, resulting in a positive resilience outcome [16]. In this study, some working mothers dynamically interacted with the environment to take actions by using problem-solving skills. Through constructing a high-support, low-stress breastfeeding environment, these mothers maintain to breastfeed and achieve natural weaning. The process of natural weaning shows the gentle and positive weaning experience both for mothers and babies, which means the resilience reconstruction may be a mental balance and mutual process driven by both mother and baby [15, 38]. This new point may need further researches to discuss. Although WHO recommended continued breastfeeding for 2 years or more [1] and some anthropologists thought the natural age of weaning for children is between 2.5 and 7 years from the biological view [43]. In this study, natural weaning occurs before the child is 2 years old and mother and baby experience a mutual interaction. Johnsen (2002) also believes that optimal weaning depends on the needs and wants of both the mother and baby [14]. Thereby, further research may be needed to explore the relationship between natural weaning and the duration of breastfeeding.

Active weaning is a type of adaptation. Adaptation is defined as individuals return to the prestress state with average resilience [16]. In this study, some working mothers set a goal for breastfeeding timeframe. They tried to improve their environment, gain support, and overcome challenges during breastfeeding in the phases of behavioral resilience. Once they had reached their goals, they wanted to resume work as soon as possible in face of new stressors. In such cases, it was difficult for them to continue to build resilience and facilitate behavior changes. The dynamic interaction had to be stopped, resulting in active weaning in which the child’s mental needs may or may not have been met. In our study, one working mother ended breastfeeding after she reached her breastfeeding goal. But she was hesitant, because she found her child was not ready for weaning and worried about if her baby could make emotional and physical adjustment to this transition. It suggested that further researches are needed on exploring targeted interventions to help these mothers return to breastfeeding again.

Forced weaning is a type of maladaptive reconstruction. Maladaptive reconstruction is defined as a negative interaction between individual resilience and the environment, resulting in negative resilience outcome [16]. During the phases of behavioral resilience, although some employed mothers had taken efforts to overcome the difficulties, they faced overwhelming new stressors (such as severe cervical disease), which along with a lack of strong support and stress management skills. They may cause forced weaning suddenly. Sudden weaning is usually painful for mother and difficult for baby [42]. One of the mothers in this study often feel “guilty”. Both the mother and the baby have unmet mental and emotional needs. Forced weaning as the outcome of negative resilience may result in the negative experience and feeling of weaning both for mothers and their babies. It is necessary to study how to avoid negative resilience outcome and what the beneficial ways are to make weaning a positive experience for employed mother with negative resilience and their children. Additionally, the forced weaning may be culturally different. The forced weaning of working women in this study may be related to their inability to balance the changing pressure and support. Johnsen’s research suggested that women’s forced weaning was closely related to low-income and different races to obtain social support [14]. This showed the different social and cultural backgrounds of forced weaning between different countries.

This study identified three different weaning processes as the resilience outcomes. Although previous studies considered that weaning is the transition of infant feeding from mother’s milk to complete reliance on other food [44], our study suggested weaning processes also involved mental, emotional and social adjustment and interactions between mothers and babies because of the dynamic interaction between resilience and environment during breastfeeding. That may provide new ideas for researches on promoting the positive and gentle weaning experience for both the mother and child.

## Limitations

Two researchers evaluated and analyzed the data in this study, and different methods were used to improve the data reliability. However, the results may not be objective, as they may inevitably reflect the authors’ ideas and opinions. Moreover, the employed mothers enrolled in this study worked in the public services with stable welfare, for instance as teachers, healthcare professionals, policewomen, government officers, and designers. Their common feature is not only that they have stable jobs, but also that they are well-educated women. Future research are needed to look at working mothers who did not go through higher education or without stable jobs.

## Conclusions

In this study, we used Kumpfer’s Resilience Framework to form the framework of resilience in employed mothers who were in the adversity of breastfeeding after returning to work. This framework shows that the transactional processes between environmental factors (stressors, supports) and individuals stimulates resilience qualities in working mothers, and then dynamic interaction between resilience qualities and environment leads to behavioral resilience, the ongoing dynamic interaction between behavioral resilience and environment leads to three different weaning processes. The findings provide new insights into the resilience of employed mothers around the world to continue breastfeeding and show the different culture of breastfeeding on employed mothers.

It is suggested by the researchers that the resilience framework emerged from this study could be a helpful guide for employed mothers returning to work to consider how they interact with environmental factors to build resilience qualities and lead to positive resilience outcomes so that they may insist on breastfeeding positively. It is valuable for midwifery practices to help employed mothers who in the adversity of breastfeeding to build and improve positive resilience. It also may provide reference for workplaces and governments when developing policies related to supporting breastfeeding women after returning to work.

## Data Availability

The dataset or transcripts are available from the corresponding authors upon reasonable request.

## Abbreviations

CDRF: China Development Research Foundation
WHO: World Health Organization

## Footnotes

1 weaning processes: The word “weaning” refers to cessation of breastfeeding.
